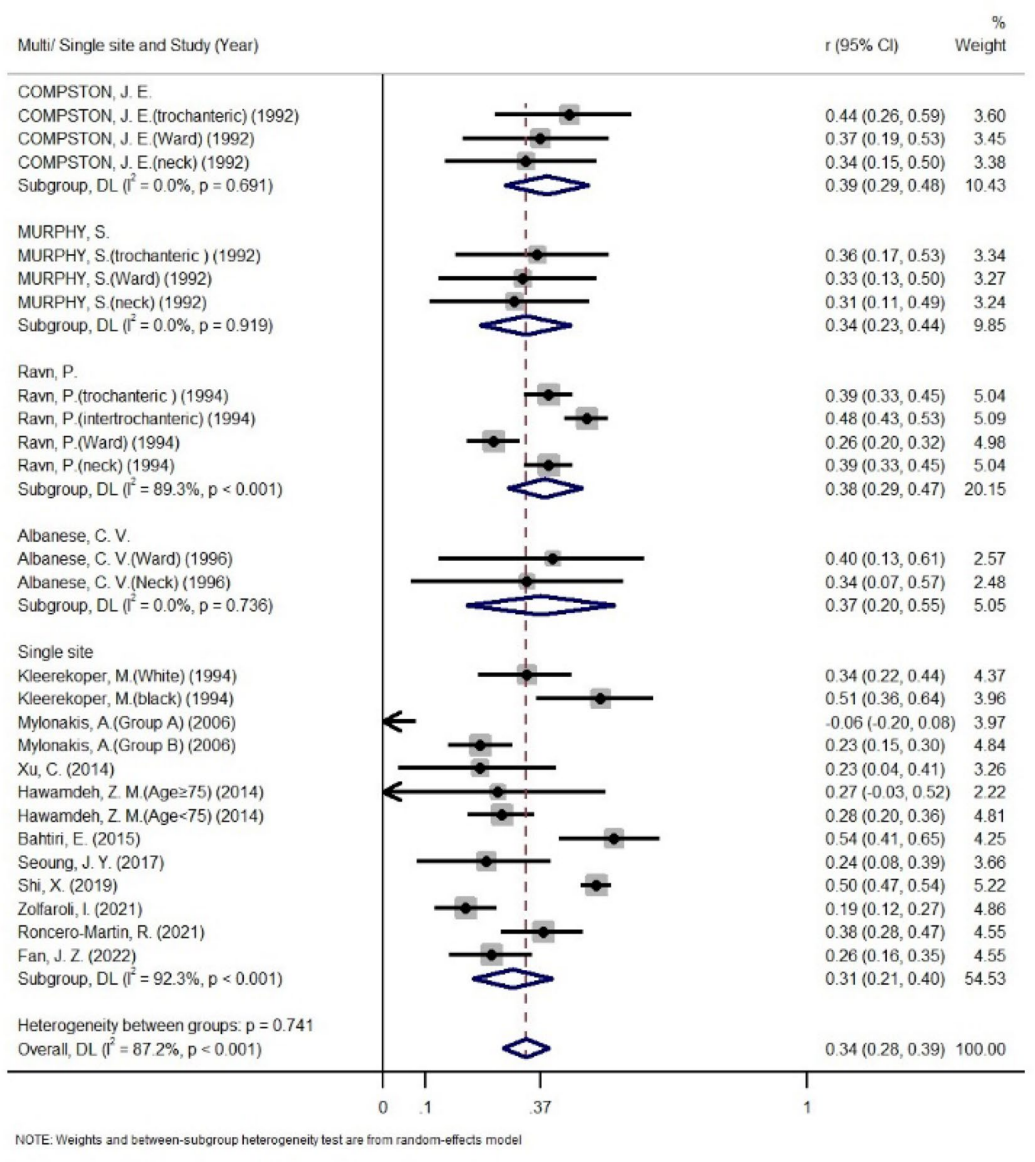# Correction

**DOI:** 10.1080/07853890.2025.2571256

**Published:** 2025-10-09

**Article title:** Association of body mass index and bone mineral density in postmenopausal women: a systematic review and meta-analysis

**Authors:** Cai, S., Gao, J., Li, C., Lu, F., Hu, D., Luo, X., Zhao, Z., Pan, Y., and Duan, G.

**Journal:** *Annals of Medicine*

**Bibliometrics:** Volume 57, Number 01, pages 25612227

**DOI:** https://doi.org/10.1080/07853890.2025.2561227

When this article was first published online, Figure 5D contained incorrect details. The corrections are as follows:

1. “*B and Study (Years)*” has been revised to: “*Multi/Single site and Study (Year)*”
2. “G” has been revised to: “*Single site*”
3. The order of subgroups (*Single site* and *Albanese, C.V.*) has been rearranged for improved visualization.

These corrections have now been implemented, and the figure has been republished with the correct details. The updated Figure 5D is attached below.

Figure 5D

**Figure:**